# Ultrafast and Cost-Effective Pathogen Identification and Resistance Gene Detection in a Clinical Setting Using Nanopore Flongle Sequencing

**DOI:** 10.3389/fmicb.2022.822402

**Published:** 2022-03-17

**Authors:** Ekaterina Avershina, Stephan A. Frye, Jawad Ali, Arne M. Taxt, Rafi Ahmad

**Affiliations:** ^1^Department of Biotechnology, Inland Norway University of Applied Sciences, Hamar, Norway; ^2^Division of Laboratory Medicine, Department of Microbiology, Oslo University Hospital, Oslo, Norway; ^3^Faculty of Health Sciences, Institute of Clinical Medicine, UiT - The Arctic University of Norway, Tromsø, Norway

**Keywords:** ONT sequencing, pathogen identification, antibiotic resistance gene (ARGs), clinical sample, Flongle

## Abstract

Rapid bacterial identification and antimicrobial resistance gene (ARG) detection are crucial for fast optimization of antibiotic treatment, especially for septic patients where each hour of delayed antibiotic prescription might have lethal consequences. This work investigates whether the Oxford Nanopore Technology’s (ONT) Flongle sequencing platform is suitable for real-time sequencing directly from blood cultures to identify bacteria and detect resistance-encoding genes. For the analysis, we used pure bacterial cultures of four clinical isolates of *Escherichia coli* and *Klebsiella pneumoniae* and two blood samples spiked with either *E. coli* or *K. pneumoniae* that had been cultured overnight. We sequenced both the whole genome and plasmids isolated from these bacteria using two different sequencing kits. Generally, Flongle data allow rapid bacterial ID and resistome detection based on the first 1,000–3,000 generated sequences (10 min to 3 h from the sequencing start), albeit ARG variant identification did not always correspond to ONT MinION and Illumina sequencing-based data. Flongle data are sufficient for 99.9% genome coverage within at most 20,000 (clinical isolates) or 50,000 (positive blood cultures) sequences generated. The SQK-LSK110 Ligation kit resulted in higher genome coverage and more accurate bacterial identification than the SQK-RBK004 Rapid Barcode kit.

## Introduction

The rising antimicrobial resistance of recent years poses a major threat to humanity and rapid diagnostic tools for bacterial infections are urgently needed (Avershina et al., 2021a). The current state-of-the-art in infection diagnostics are mostly based on biochemical analysis of cultured clinical samples (Vasala et al., 2020). The turnaround time is around 2–4 days for most samples, but a longer time is required for low bacterial load samples, such as bloodstream infection (BSI) [usually 1–100 colony-forming units (CFU)/mL], where identification and antibiotic susceptibility testing (AST) could take up to 5 days (Metzgar et al., 2016; Briggs et al., 2021). There are emerging micro- and nanotechnologies for bacterial identification and AST, including both phenotypic (e.g., microfluidic-based bacterial culture) and molecular (e.g., multiplex PCR, hybridization probes, nanoparticles, synthetic biology, and mass spectrometry) methods (Li et al., 2017).

The very limited availability of rapid, easy-to-use and scalable methods to interpret whole-genome sequencing (WGS) data for clinical purposes make it challenging. Illumina sequencing is not time-efficient, but recent studies have shown that Oxford Nanopore Technology’s (ONT) MinION could potentially be used for point of care sequencing and become a basis for WGS-based diagnostic strategies (Harstad et al., 2018; Taxt et al., 2020). Examples of its clinical usefulness include same-day diagnostic results for tuberculosis and blood cultures (Votintseva Antonina et al., 2017; Taxt et al., 2020). Also, bacterial pathogen identification in lower respiratory infection (sputum samples) within 6 h has been demonstrated (Charalampous et al., 2019).

The faster a resistance profile of an infectious agent is known, the faster the right treatment can be initiated (Trevas et al., 2021). We have previously demonstrated that MinION real-time sequencing can be successfully used for bacterial identification and antimicrobial resistance (AMR) detection in blood cultures within 4 h after the blood culture was flagged positive (Taxt et al., 2020). Flongle provides a less expensive sequencing setup than MinION (512 sequencing channels) but it has a lower total data output as it only has a maximum of 126 available sequencing channels, i.e., pores, with 60 guaranteed by the manufacturer (Grädel et al., 2019).

There is currently a very limited number of publications using Flongle flow cell sequencing available at PubMed. A search on August 16, 2021, with keywords “Flongle” and “nanopore Flongle,” resulted in a total of only 14 publications. With regard to its use in clinical microbiology, a Flongle-based assay has produced accurate and reproducible results for enterovirus identification and genotyping (Grädel et al., 2019). Also, recently Flongle and MinION have been used in profiling preterm microbiota and AMR in fecal samples (Leggett et al., 2020). However, only 1 out of 20 samples was sequenced with the Flongle flow cell (Leggett et al., 2020), which is too small of an amount to conclude on the clinical usefulness of Flongle.

This work assessed whether Flongle flow cells are as proficient for bacterial ID and antimicrobial resistance genes (ARGs) detection as MinION flow cells. We demonstrate the performance of Flongle in the sequencing of (a) cultured clinical isolates of *E. coli* and *K. pneumoniae*, (b) blood cultures spiked with clinical isolates using Flongle flow cells with 59–60 active pores, and (c) as low as 24–39 active pores. The Flongle data were validated using previously published MinION and Illumina MiSeq sequencing data for these isolates (Taxt et al., 2020; Avershina et al., 2021b; Khezri et al., 2021a,b). We demonstrate the sufficiency of Flongle for bacterial ID and ARG detection within the first obtained sequences both on pure isolates and direct blood cultures. We also demonstrate that whole-genome sequencing is preferable over plasmid isolation and sequencing. Flongle accuracy was, however, insufficient for correct CTX-M variant calling, but this task is relatively less critical for proper antibiotic treatment adjustment.

## Materials and Methods

### Ethics Statement

In this study, we worked with bacterial isolates from the strain collection at the Department of Microbiology at Oslo University Hospital (OUH). Human blood was obtained from anonymous healthy donors via the blood bank at OUH. There was no intention to sequence human DNA and, therefore, QIAamp BiOstic Bacteremia DNA Kit for extraction of bacterial DNA was used. Moreover, any sequencing reads recognized as being generated from human DNA were omitted from further analysis and permanently discarded. Ethical approval was not deemed necessary. The clinical microbiology laboratory at OUH is approved for the described experimental work and carries out diagnostic work in the clinical routine.

### Dataset Description

This study used two clinical isolates of *Escherichia coli* (*E. coli* 125 and *E. coli* A2-39) and two clinical isolates of *Klebsiella pneumoniae* (*K. pneumoniae* 225 and *K. pneumoniae* A2-37). The rationale for using these isolates is an extensive amount of sequencing data (both Illumina MiSeq and MinION) available from our previously published works (Taxt et al., 2020; Avershina et al., 2021b; Khezri et al., 2021b). These isolates were subject to whole genome sequencing and plasmid sequencing from the bacterial culture using Flongle flow cells. Sequencing was performed both using ONT Rapid Barcoding kit SQK-RBK004 (four isolates per flow cell) and Genomic DNA by Ligation SQK-LSK110 (one isolate per flow cell). In addition, we have spiked blood with the *E. coli* A2-39 and *K. pneumoniae* A2-37 isolates, cultured overnight, and performed WGS directly on the DNA isolated from the blood culture using flow cells with two times the difference in the number of active sequencing pores. The QIAamp BiOstic Bacteremia DNA Kit from Qiagen (Germany) was used for the extraction of bacterial DNA to be used for nanopore sequencing (Taxt et al., 2020). DNA extraction was performed according to the manufacturer’s instructions. Human blood was obtained from healthy anonymous donors via the blood bank at OUH. The blood was used before the “best by” date, no later than 3 weeks after the blood was drawn. All experiments, as well as ARGs possessed by the isolates, are reported in Supplementary Table 1.

### Sample Preparation

Genomic DNA was isolated from plate-grown bacterial cultures using the Midi format of the protocol for Gram-negative and some Gram-positive bacterial samples with Genomic-tip 100/G columns following the manufacturer’s instructions (Qiagen, Germany). Plasmid DNA was isolated using the Qiagen QIAprep Spin Kit. Purified DNA was stored in Tris buffer at −20°C and quantified using the Qubit dsDNA HS assay kit (Thermo Fisher Scientific, United States). Sequencing samples were prepared for the run on Flongle flowcells following the manufacturer’s instructions. Rapid Barcoding Sequencing (SQK-RBK004) and Genomic DNA by Ligation (SQK-LSK110) kits were used together with the solutions from the custom kit FLP003. Sequencing was done on Flongle flow cells (R9.4.1) for at least 24 h. The availability of sufficient active pores was confirmed on arrival and directly before the sequencing run. The starting bias voltage was −180 mV with 1.5 h between channel scans. High accuracy base calling with Guppy version 4.3.4 run on GPU was performed. In the case of SQK-RBK004, reads were also demultiplexed at this step. The Qscore limit was 7 and no filtration for length was applied. The output was sent to *fastq* files with 1,000 reads per file. Reads recognized as generated from human DNA were omitted from further analysis and discarded.

### Whole-Genome Sequencing Data Analysis

WGS reads from each *fastq* file, as well as combined reads from each whole sequencing run, were mapped to previously published Illumina-MinION hybrid assemblies (Taxt et al., 2020; Khezri et al., 2021b; (a) of the corresponding isolates using CLC Genomic Workbench v.20.0.4 (Qiagen, Germany). Each output file was searched with Blastn v 2.6.0 against NCBI RefSeq (version June 2021) and ResFinder (version June 2021) databases for bacterial ID and ARG, respectively. Plasmids were searched against the PLSDB database using a minimum identity of 90% as a cut-off (Galata et al., 2019).

WGS data with > 100x genome coverage was split into 12 subsets. Each subset was then filtered by length (1,000 bp) using Filtlong v.0.2.1 and assembled using Flye v2.8.3, Miniasm-0.3, or Raven v1.5.1, and resulting assemblies were reconciled and optimized using Trycycler v.0.5.0.

### Plasmid Data Analysis

Reads generated from plasmid sequencing were mapped to reference hybrid assemblies (Taxt et al., 2020; Khezri et al., 2021b) using CLC Genomic Workbench, v.20.0.4 (Qiagen, Germany) and assembled using Unicycler v0.4.9 and Flye v2.8.3. Assembly graphs were visualized using Bandage v0.8.1.

Unless stated otherwise, all statistical analyses were performed in MATLAB R2020b (MathWorks Ltd., United States).

## Results

### General Statistics of the Oxford Nanopore Technology’s Flongle Runs

The number of active pores on the Flongle cells remained stable during storage in the cold room four of which lasted up to 8 months (Supplementary Table 2).

On average, 4,430 ± 3,506 [mean ± standard deviation] and 151,435 ± 95,325 reads per sample were generated using SQK-RBK004 and SQK-LSK110 kits, respectively (Table 1). N50 values comprised 9,188 ± 6,758 bp for SQK-RBK004 and 11,867 ± 5081 for SQK-LSK110, longest generated reads ranged from 24,745 to 124,329 bp for SQK-RBK004 and from 40,760 to 146,931 bp for SQK-LSK110. The total length generated for clinical isolates using the SQK-LSK110 kit was on average 126x higher compared to SQK-RBK004 (*p* < 0.05). When using SQK-RBK004, only one output file was generated after the whole sequencing was run for one of the isolates, i.e., it required > 24 h to get data for that isolate. Additionally, 1.2% of the barcodes were misidentified during the demultiplexing step.

**TABLE 1:** General statistics of the ONT Flongle runs.

Exp Nr	ID	Sample type	ONT kit	Number of active pores	Number of reads	Total length (Mbp)	Longest read (bp)	N50 (bp)
1	*E. coli* 125	Clinical isolates	SQK-RBK004	69	7,840	25.6	89,995	7,956
	*K. pneumoniae* 225				9,493	76.6	124,329	21,552
	*E. coli* A2-39				879	1.9	58,672	11,868
	*K. pneumoniae* A2-37				6,744	35.1	136,325	14,239
2	*E. coli* 125		SQK-LSK110	70	10,5368	683.5	122,281	13,232
3	*K. pneumoniae* 225			60	102,714	904.0	127,460	21,738
4	*E. coli* A2-39			67	313,395	861.6	124,559	9,871
5	*K. pneumoniae* A2-37			59	193,000	1082.9	146,931	14,684
6	*E. coli* 125	Plasmid preparation	SQK-RBK004	59	1,144	1.7	24,745	1,626
	*K. pneumoniae* 225				189	0.8	44,111	10,015
	*E. coli* A2-39				3,575	6.1	51,440	2,359
	*K. pneumoniae* A2-37				5,579	12.3	54,931	3,889
7	*E. coli* A2-39	Spiked blood culture	SQK-LSK110	62	293,814	1273.8	40,760	7,044
8	*K. pneumoniae A2-37*			59	127,537	756.8	41,029	7,961
9	*E. coli* A2-39			39	95,689	528.6	44,214	7,260
10	*K. pneumoniae A2-37*			24	93,892	583.5	39,975	8,339

### First Output Files of Whole-Genome Sequencing Are Sufficient for Bacterial Identification and Antimicrobial Resistance Gene Detection in Clinical Isolates

**FIGURE 1:**
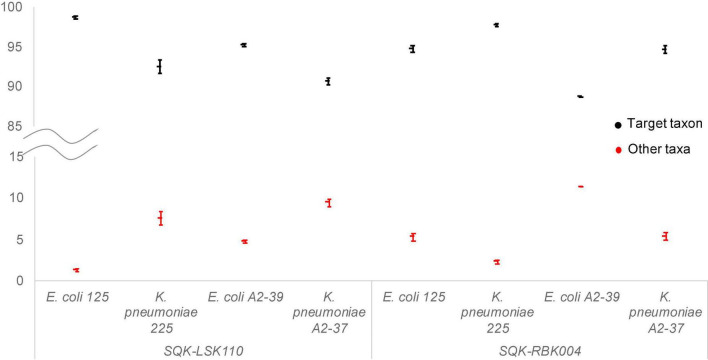
Average relative abundance of target taxa (black) and other non-target taxa (red) throughout ONT Flongle sequencing runs. Error bars represent the standard deviation of the relative abundance with every 1,000 sequences added.

Taxonomic assignment of reads remained the same throughout the whole sequencing run (Figure 1), indicating that the first generated and outputted 1,000 sequences were as sufficient for bacterial identification as the data from the whole sequencing run. *K. pneumoniae* reads in A2-37 (experiments 1 and 5) were commonly misclassified as *K. quasipneumoniae*—the species that is closely related to *K. pneumoniae* (Long et al., 2017). In *E. coli* A2-39 (SQK-RBK004, experiment 1), only 189 sequences were generated, and nine percent of these reads were assigned to *K. pneumoniae*. Interestingly, there was no misidentification of *E. coli* to *K. pneumoniae* using SQK-LSK110 (experiment 4).

First hits to ARGs were found in the first or the second generated output file (5 min to 2.6 h after the sequencing start), and the whole resistome was detected at most within the first 3,000 sequences. However, assignment of the correct variant (SHV-187, CTX-M-2, CTX-M-14) was not possible using single Flongle reads (Supplementary Table 3).

For *E. coli* 125, the target gene TEM-1B was not detected either when the isolate was sequenced using SQK-RBK004 [experiment 1; WGS data map to 75% of the reference hybrid assembly (Khezri et al., 2021b) with 3X coverage] or using Ligation kit [experiment 2, WGS data map to 99% of the reference hybrid assembly (Khezri et al., 2021b) with 132X coverage]. Interestingly, the TEM-1B gene in this isolate was also previously not detected by the MinION, although confirmed by Illumina sequencing (Khezri et al., 2021b). In the reference hybrid assembly, TEM-1B was located on a 10,909 bp plasmid contig (99.9% identity to *E. coli* O55:H7 strain RM12579 p12579_5; 5954 bp). This contig was supported only by the Illumina data (4,750 reads mapped, 27x coverage) and additionally contained Tn3 family transposase Tn2, Transposon Tn3 resolvase, and DNA relaxase MbeA (Supplementary Table 4). Neither Flongle nor previously published MinION generated reads (Khezri et al., 2021b) mapped to this contig. Coverage of the TEM-1B gene by Illumina data ranged from 7X to 25X. We then performed PCR to address the discrepancy between the platforms (Supplementary Text 1). The presence of TEM-1 was not supported by PCR (Supplementary Figure 1).

### SQK-LSK110 Flongle Generated Data Is Sufficient for Whole-Genome Coverage of the Clinical Isolates Using at Most the First 20,000 Sequencing Reads Generated

Due to the large variation in time required for the generation of each 1,000 sequences [around 10–30 min per 1,000 sequences when using one sample per sequencing run (SQK-LSK110) and up to 6 h when using four samples per run (SQK-RBK004)], we decided to report the data with regards to the number of sequences generated rather than an actual timeline.

**FIGURE 2:**
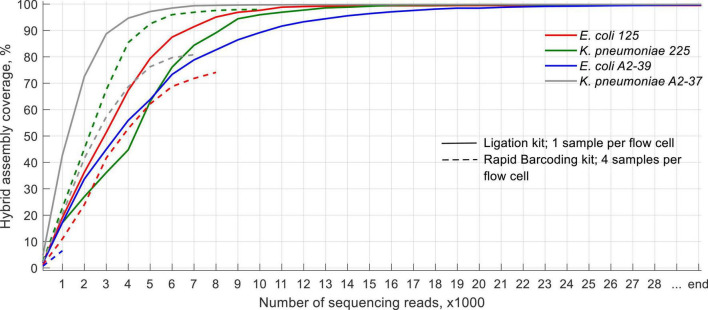
Coverage of the reference hybrid assemblies throughout the Flongle sequencing run.

With SQK-RBK004, only the *K. pneumoniae* 225 isolate had a sufficient amount of sequencing data to cover > 95% of its reference hybrid assembly at least once by the end of the run (experiment 1, Figure 2). For the other isolates, however, portions of genome sequenced ranged from < 10% (*E. coli* A2-39, experiment 4) to 80% (*K. pneumoniae* A2-37, experiment 5). When using the ligation kit, all isolates covered > 99% reference hybrid assembly within at most the first 20,000 sequences. On average, these data were generated 4.5 h after the sequencing started.

### The Flongle Based Assemblies of Clinical Isolates Are Not Suitable for Antimicrobial Resistance Gene Variant Calling

**FIGURE 3:**
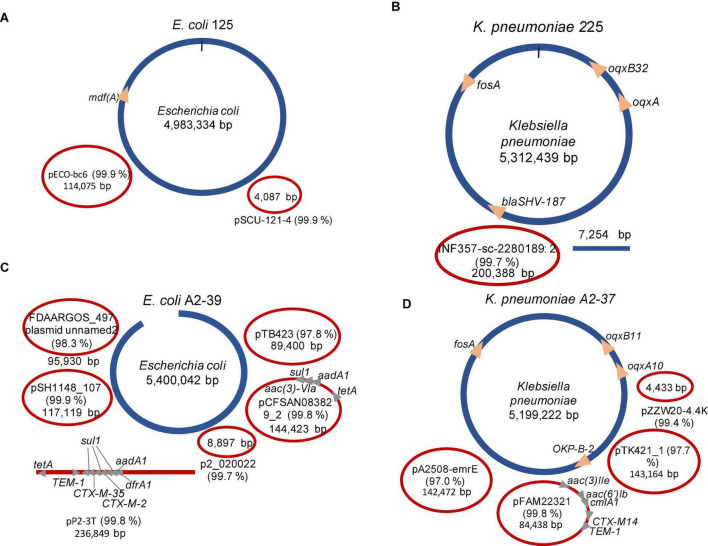
Summary of the Trycycler assemblies’ reconciliation of clinical isolates using SQK-LSK110 Flongle. Blue color depicts chromosomal contigs, red color—plasmid contigs. Circular contigs are represented as closed ellipses, linear—as lines, and open ellipses. For plasmid contigs, the closest search hit against the PLSDB plasmid database is given as a plasmid name. Identity% is provided in brackets. **(A)** *Escherichia coli* 125 (experiment 2); **(B)** *Klebsiella pneumoniae* 225 (experiment 3); **(C)** *Escherichia coli* A2-39 (experiment 4); **(D)** *Klebsiella pneumoniae* A2-37 (experiment 5).

On average, Flye, Miniasm and Minipolish, and Raven assembled each isolate (experiments 2–5) into 6 ± 4 contigs. After clustering and assemblies’ reconciliation, the number of contigs on average reduced to 4 ± 2 (Figure 3), and chromosomes were fully assembled. In the case of *K. pneumoniae* 225 (experiment 3) and *E. coli* A2-39 (experiment 4), target ARGs were correctly assigned to an expected variant, SHV-187, and CTX-M-2, respectively (Figure 3). However, unlike reference hybrid assembly (Khezri et al., 2021b), Flongle assembly suggests that SHV-187 was located on the *K. pneumoniae* 225 chromosome rather than on a plasmid. It is worth noting that using Trycycler assembly, an additional CTX-M-35 was detected on the same plasmid from *E. coli* A2-39 isolate upstreams of CTX-M-2. Twenty-four raw Flongle reads (8,816–71,122 bp long) had an additional CTX-M gene located approximately (≈)4 kbp downstream from the first CTX-M gene. Both copies had 35–39x coverage by MinION reads, but none of the reads covered two copies simultaneously. Illumina reads supported only CTX-M-2 (73X), but not CTX-M-35.

Interestingly, one of the plasmids in *E. coli* A2-39 isolate (experiment 4), where 2.3% reads were assigned to *Salmonella enterica* (Figure 1), was closely related to *Salmonella enterica* subsp. *enterica* plasmid pSH1148_107 (Figure 3).

### Flongle Sequencing of Plasmids From Clinical Isolates Recovered < 9 kbp Long Plasmids Only

On average, 2,622 ± 2,433 reads per isolate were generated using the SQK-RBK004 kit (experiment 6, Table 2). In the *K. pneumoniae* 225 isolate, a single output file with 189 reads was generated. The majority of reads mapped to chromosomal contigs of the reference hybrid assembly (Supplementary Table 5), and only 9.3% (*K. pneumoniae* 225) to 37.6% (*E. coli* A2-39) mapped to plasmid contigs. A minor part of generated reads could be assembled into circular or linear contigs of at most 16,324 bp (Table 2). Plasmid sequencing only revealed < 9 kbp long plasmids from the isolates. Interestingly, for *K. pneumoniae* A2-37, Unicycler produced one circular plasmid of 4,447 bp closely related to *K. pneumoniae* plasmid pZZW20-4.4K, 4,436 bp, also detected using WGS Flongle sequencing of the clinical isolate (experiment 5). Conversely, Flyeproduced three contigs. Two of these contigs gave the closest PLSDB match to plasmids 4–10 times shorter, including a match to *K. pneumoniae* plasmid pZZW20-4.4K. The third contig did not produce any close match to a plasmid using PLSDB (Table 2).

**TABLE 2:** Summary of ONT Flongle sequencing of plasmids.

Plasmids from	Total number of reads generated	Longest raw read, bp	Assembly				Closest match at PLSDB					Target AMR	
			Assembler	Assembly length, bp	Assembly graph	Raw reads mapped to assembly	Plasmid ID	Plasmid length, bp	ID,%	AMR	Assembled in WGS	Raw reads	Assembly
*E. coli* 125	1,144	24,745	Unicycler	5,644	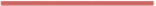	21	*E. coli* pSCU-121-4; CP054332.1	4,091	99.9	No	Yes	No	No
			Flye	4,083	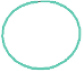	20	*E. coli* pSCU-121-4; CP054332.1	4,091	99.8	No	Yes		No
*K. pneumoniae* 225	189	44,111	Unicycler	3,295	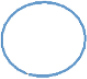	43	*K. pneumoniae* INF072-sc-2279995, plasmid: 4; LR890188.1	3,302	99.8	No	No	No	No
			Flye	Too little data, error in the assembly								No	−
*E. coli* A2-39	3,575	51,440	Unicycler	8,893	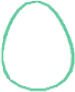	102	*E. coli* p2_020022; NZ_CP032881.1	8,899	99.7	No	Yes	No	No
			Flye	7,655	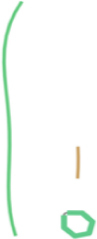	149	*E. coli* p2_020022; NZ_CP032881.1	8,899	98.5	No	Yes		No
				2,347			*E. coli* pSCU-172-7; NZ_CP054360.1	2,311	99.7	No	No		No
				1,033			no hits	−	−	−	−		No
*K. pneumoniae* A2-37	5,579	54,931	Unicycler	4,447	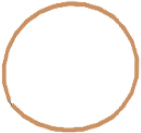	288	*K. pneumoniae* pZZW20-4.4K; NZ_CP058963.1	4,436	98.8	No	Yes	CTX-M, 1st file	No
*K. pneumoniae* A2-37	5,579	54,931	Flye	16,324	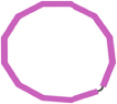	911	*Klebsiella pneumoniae* plasmid pZZW20-4.4K; NZ_CP058963.1	4,436	96.2	No	Yes	CTX-M, 1st file	No
				13,264	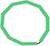		*Klebsiella grimontii* SS141 plasmid_3.2; NZ_CP044531.1	1,180	96.2	No	No		No
				4,519	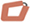		−	−	−	−	−		No

The CTX-M gene was detected in a single read (14,495 bp) from *K. pneumoniae* A2-37 plasmid data (CTX-M, variant not assigned) (Table 2). After assembly, however, the CTX-M gene was not detected in any of the contigs. Based on the WGS data from the clinical isolate (experiment 5), the gene was located on the circular 84,438 bp plasmid closely related to *K. pneumoniae* pFAM22321 (Figure 3). Indeed, the 14,495 bp CTX-M containing read mapped to this plasmid contig with 87% coverage.

### Direct Flongle Sequencing of Positive Blood Cultures Is as Efficient as That of the Pure Clinical Isolates for Bacterial ID and Antimicrobial Resistance Identification, but Not for Plasmid Detection

Same as with clinical isolates, the first file out was sufficient for bacterial ID as any other consequent file, albeit with a higher fluctuation in relative abundances (Supplementary Figure 2). Human reads comprised < 10% in all cases apart from *K. pneumoniae* A2-37 blood culture, where human data comprised 20% of the sequencing data. Like *K. pneumoniae* A2-37 clinical isolate sequencing, reads were also assigned to *K. quasipneumoniae*.

**FIGURE 4:**
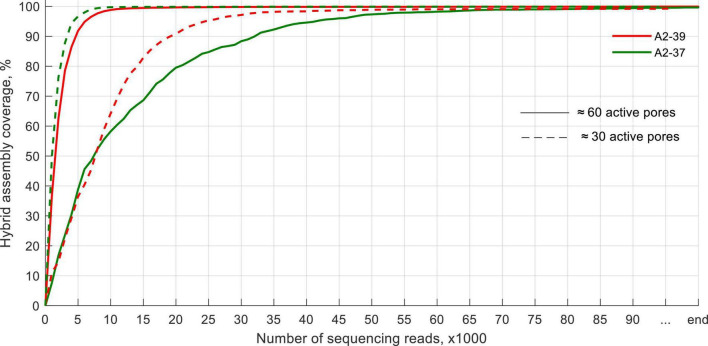
Coverage of the genome by ONT Flongle sequencing of the spiked blood cultures.

Using a flow cell with ≈60 active pores required at most 75,000 reads to sequence > 99% of the genome at least once for the blood culture spiked with *K. pneumoniae* A2-37 (Figure 4). Using a flow cell with ≈30 active pores did not impair the sequencing performance. It took, at most, 56,000 reads to sequence > 99% of the genome, and in this case, *E. coli* A2-39 blood culture required more sequencing data. All ARGs were detected within the first 3,000–4,000 sequences apart from the sample spiked with *K. pneumoniae* A2-37 and sequenced using a flow cell with ≈60 active pores. In that case, the first indication of *fosA* and *oqxA* genes appeared only after 10,000 reads. Same as with sequencing of pure clinical isolates, ARG variant detection was not possible.

Comparison of plasmid contigs between assemblies from clinical isolates and blood cultures of these clinical isolates revealed 99.7–99.9% pairwise identity between the corresponding contigs (Supplementary Table 6). However, blood culture sequencing assemblies of *E. coli* A2-39 missed a plasmid closely related to *Salmonella enterica* subsp. *enterica* plasmid pSH1148_107 (NC_019123.1). Both *K. pneumoniae* A2-37 blood culture assemblies lacked a plasmid contig closely related to *K. pneumoniae* plasmid pZZW20-4.4K, 4436 bp (NZ_CP058963). Same as with clinical isolate assembly, CTX-M-14 was found located on a plasmid contig in *K. pneumoniae* A2-37. In the case of *E. coli* A2-39, two copies of the CTX-M-35 gene were detected on plasmid contigs. Twelve raw Flongle reads contained both copies of the CTX-M gene, separated by ≈ 4 kbp region.

## Discussion

Fast identification of the infection is crucial for proper treatment. Previously we have established that real-time ONT MinION sequencing allows rapid identification of bacteria within 10 min after the sequencing run (Taxt et al., 2020). In this work, we demonstrate that a cheaper Flongle setup is also sufficient for fast detection and identification of bacteria, especially using one sample per flow cell. The first output files of 1,000 sequences produced enough data to identify target bacteria both when pure isolates and spiked blood cultures were sequenced, albeit the time needed to generate the first file varied extensively between flow cells and sequencing kits. When using the ligation kit, the first 1,000 sequences were available in 10–15 min after the sequencing started. Conversely, the rapid barcoding kit required up to 2 h to generate the first output file for each barcode. Additionally, we noticed a possible miscalling of used barcodes. A study conducted on MinION sequencing of three viruses suggested around 0.056% of the generated reads to be misassigned to a different barcode (Xu et al., 2018). In our work, Flongle sequencing has exhibited a higher rate of barcode misassignment, with 1.2% of generated reads assigned to barcodes that were not used in the run using standard Guppy base calling and demultiplexing provided by ONT. Therefore, we suggest that it is safer to sequence one sample per flow cell during clinical sample sequencing.

We observed high variation between throughput for each isolate when using rapid barcoding, with some genomes being only < 10% sequenced throughout the whole run. When one sample per flow cell was sequenced, conversely, the total throughput was > 100× genome coverage, and around 20,000–50,000 generated sequences covered the whole genome to be sequenced at least once.

The first 3,000 sequences were sufficient for detecting target ARGs both when sequencing clinical isolates and blood cultures spiked with these isolates in all instances. Same as with MinION, however, variant detection was not possible until the data were assembled (Taxt et al., 2020). Flongle generated sequencing data previously exhibited high concordance with Illumina data regarding gene fusion detection (Jeck et al., 2021). In our work, however, there were discrepancies between ONT flow cells and Illumina MiSeq generated data, especially regarding ARG content. Unlike MinION and MiSeq, Flongle sequencing data found the CTX-M-35 gene in addition to the CTX-M-2 gene in the *E. coli* A2-39 plasmid. These two copies were separated by a ≈4 kbp region which was supported by a total of 36 raw Flongle reads.

The other discrepancy between ONT (both MinION and Flongle) and Illumina MiSeq data with regards to ARGs is the TEM-1B penicillinase in *E. coli* 125. Phenotypically, *E. coli* 125 was susceptible to penicillin (Avershina et al., 2021b), which suggests that the TEM-1B gene in this isolate was not expressed. Due to numerous rounds of PCR, Illumina sequencing data are prone to chimera generation (Arroyo Mühr et al., 2020), and chimera removal is a common filtering step when using amplicon sequencing with Illumina (Prodan et al., 2020). However, it seems doubtful that a TEM-1B-identical sequence would be chimera-generated. Therefore, we believe that the absence of TEM-1B might have been caused by a loss of the TEM-1B containing plasmid by *E. coli* 125.

ARGs located on plasmids can be transferred across species and thus pose a higher threat of broad distribution than those located on a chromosome. Moreover, plasmids can be present in multiple copies in a bacterial cell, and the plasmid copy number can increase if environmental conditions favor the survival of plasmid carriers (f.ex. presence of antibiotic) (San Millan et al., 2015). It has been previously demonstrated that plasmids > 150 kbp in size are difficult to extract using column-based methods (Villa and Carattoli, 2020). WGS sequencing was recently recommended for plasmid reconstruction using ONT MinION over plasmid extraction and sequencing (Berbers et al., 2020). In our work, we also favor whole-genome sequencing to detect plasmid-borne ARGs and plasmid reconstruction with ONT Flongle.

Currently, the price per Flongle flow cell, which according to the manufacturer, can deliver up to 2.8 gigabytes of data, is $90^1^. Given its fast turn-around time, and low cost, Flongle sequencing has a high potential for being used for infection diagnostics in a clinical setting. According to ONT, the recommended Flongle flow cell storage time is 4 weeks^2^. However, we have stored the flow cells for as long as 8 months, and we observed that the number of active pores remained similar. This is a very relevant observation, which shows that the Flongle flow cells could still be used and provide sufficient data, thereby being more cost-effective. Besides, ONT is launching a ligation-based sequencing kit for multiplexing samples ($600 for 6 reactions). Based on our positive results for the ligation-based sequencing kit, this would be an interesting approach for future studies.

## Conclusion

In this work, we demonstrated that Flongle can be successfully used for the identification of bacteria and for the detection of antibiotic resistance genes both in clinical isolates and when using direct sequencing of blood spiked with these clinical isolates and cultured over-night. We suggest that currently, the optimal setup is to sequence one sample per flow cell, which provides an adequate amount of data to give the relevant information. Given that the task of whole-genome assembly is not a requirement in routine clinical diagnostics, therefore the sequencing run can be stopped after the first 20,000–50,000 sequences as with that amount of data, the whole genome was covered at least once. We also demonstrated that whole gDNA is preferable to plasmid isolation prior to sequencing since the latter poses the risk of missing ARGs. In conclusion, although the Flongle showed higher error in ARG variant calling than MinION, it still displays similar performance in real-time for pathogen ID and ARG detection. This, together with its cost-effectiveness, could lead to potential future use in clinical microbiology.

## Data Availability Statement

The datasets used and analyzed during the current study are deposited in the European Nucleotide Archive (ENA) at EMBL-EBI under accession number PRJEB49072.

## Author Contributions

EA, SF, AT, and RA designed the experiments. EA and RA wrote the manuscript. SF and JA performed experimental work in discussions with AT and RA. EA analyzed the data in discussions with RA. SF and AT edited the manuscript. All authors read and edited the manuscript.

## Conflict of Interest

The authors declare that the research was conducted in the absence of any commercial or financial relationships that could be construed as a potential conflict of interest.

## Publisher’s Note

All claims expressed in this article are solely those of the authors and do not necessarily represent those of their affiliated organizations, or those of the publisher, the editors and the reviewers. Any product that may be evaluated in this article, or claim that may be made by its manufacturer, is not guaranteed or endorsed by the publisher.

## Footnotes

1
https://nanoporetech.com/

2
https://store.nanoporetech.com/flongle-flow-cell-pack.html

## Abbreviations

AMR: antimicrobial resistance
ARG: antimicrobial resistance genes
ONT: Oxford Nanopore Technology
WGS: Whole-genome sequencing.
