# Supplementary material for: Ultrafast and Cost-Effective Pathogen Identification and Resistance Gene Detection in a Clinical Setting Using Nanopore Flongle Sequencing
Source: Front Microbiol. 2022 Mar 17;13:822402. doi: 10.3389/fmicb.2022.822402 (PMC8970966; doi:10.3389/fmicb.2022.822402)
Supplement: Supplementary file 2 [file Data_Sheet_1.docx]

**Ultrafast and cost-effective pathogen identification and resistance gene detection in a clinical setting using Nanopore Flongle sequencing**

Ekaterina Avershina^1^, Stephan A. Frye^2^, Jawad Ali^1^, Arne M. Taxt^2^, Rafi Ahmad^1,3,*^

*^1^Department of Biotechnology, Inland Norway University of Applied Sciences, Holsetgata 22, 2317, Hamar, Norway.*

*^2^Department of Microbiology, Division of Laboratory Medicine, Oslo University Hospital, PB 4956, Nydalen, 0424, Oslo, Norway.*

*^3^Institute of Clinical Medicine, Faculty of Health Sciences, UiT - The Arctic University of Norway, Hansine Hansens veg 18, 9019, Tromsø, Norway.*

*corresponding author: rafi.ahmad@inn.no; +47 62 51 78 45

**Supplementary** **Figures**

**
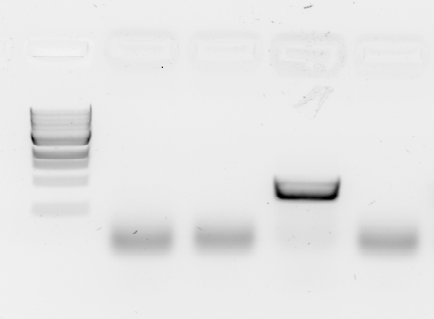
**

**1**

**2**

**5**

**3**

**1000 bp**

**847 bp**

**4**

Supplementary Figure 1. TEM-1 specific PCR. Lane 1: 1KB DNA Ladder, Lane 2: E. coli 101 (Negative control), Lane 3: E. coli 125, Lane 4: E. coli A2-39 (Positive control), Lane 5: PCR control (No DNA).


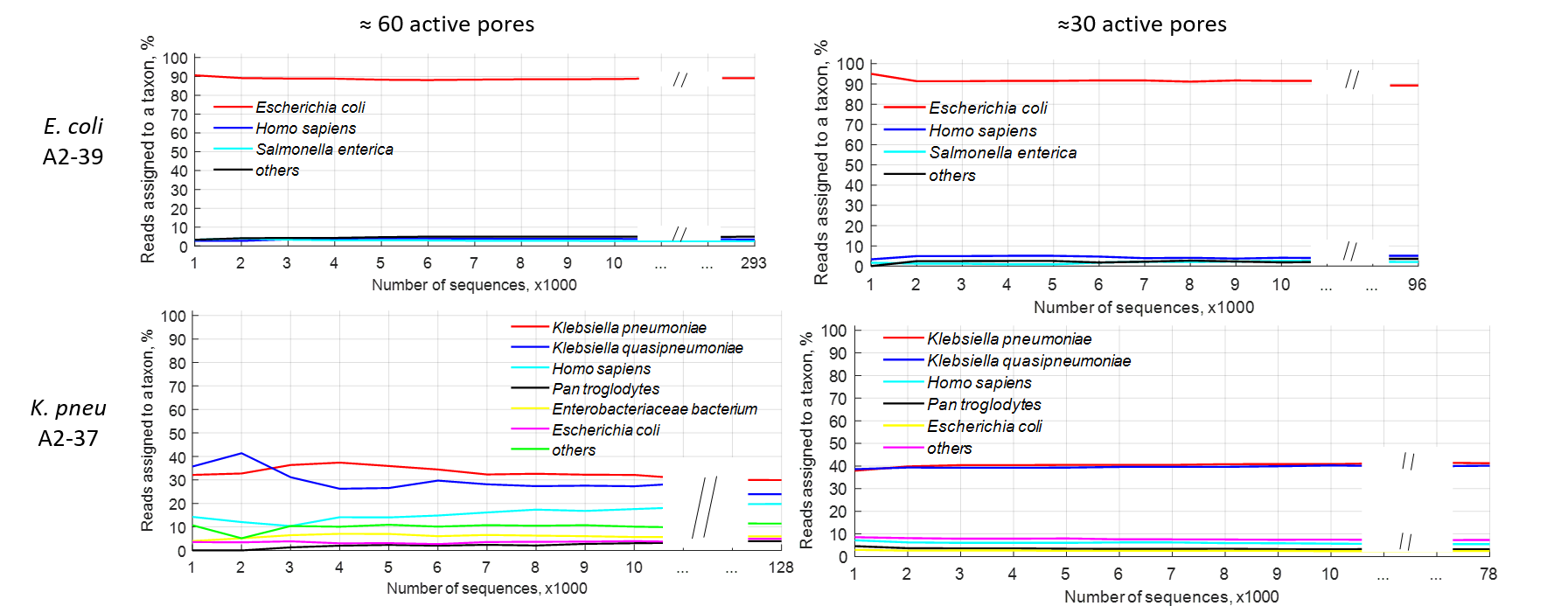


Supplementary Figure 2. Taxonomic assignment of Flongle sequencing reads from spiked blood cultures throughout the runs.
