# Supplementary material for: Ultrafast and Cost-Effective Pathogen Identification and Resistance Gene Detection in a Clinical Setting Using Nanopore Flongle Sequencing
Source: Front Microbiol. 2022 Mar 17;13:822402. doi: 10.3389/fmicb.2022.822402 (PMC8970966; doi:10.3389/fmicb.2022.822402)
Supplement: Supplementary file 3 [file Data_Sheet_2.DOCX]

**Ultrafast and cost-effective pathogen identification and resistance gene detection in a clinical setting using Nanopore Flongle sequencing**

Ekaterina Avershina^1^, Stephan A. Frye^2^, Jawad Ali^1^, Arne M. Taxt^2^, Rafi Ahmad^1,3,*^

*^1^Department of Biotechnology, Inland Norway University of Applied Sciences, Holsetgata 22, 2317, Hamar, Norway.*

*^2^Department of Microbiology, Division of Laboratory Medicine, Oslo University Hospital, PB 4956, Nydalen, 0424, Oslo, Norway.*

*^3^Institute of Clinical Medicine, Faculty of Health Sciences, UiT - The Arctic University of Norway, Hansine Hansens veg 18, 9019, Tromsø, Norway.*

*corresponding author: rafi.ahmad@inn.no; +47 62 51 78 45

**Supplementary Text**

**Supplementary Text 1. TEM gene PCR protocol**

PCR was performed by using the TEM gene specific primers (F: AGTATTCAACATTTYCGTGT & R: TAATCAGTGAGGCACCTATCTC) as described in (Copur Cicek et al., 2013). A single PCR reaction contained 2.5 µl HOTFIREPol® 10X buffer B2, 0.5 µl dNTPs, 2 µl MgCl_2_ (25mM), 1 µl of forward and reverse primers in 10mM concentration, 0.5 µl HOTFIREPol® DNA Polymerase (Solis BioDyne, Estonia) and 50ng DNA template. PCR water was added to the final volume of 25 µl. PCR cycling protocol started with initial denaturation at 95°C for 15 min followed by 45 cycles of denaturation at 95°C for 15 s, annealing at 54°C for 30 s and elongation at 72°C for 45 s, and a final extension of 72°C for 10 min. *E. coli* A2-39 isolate with confirmed TEM-1B gene (Taxt et al., 2020), was used as a positive control and *E. coli* 101 isolate which did not possess TEM-1B gene (Avershina et al., 2021), as a negative control. PCR products were run on 1% agarose gel at 100V for 30 min in 1X TAE buffer.
